# Mechanisms Behind the Inhibition of Lung Adenocarcinoma Cell by Shikonin

**DOI:** 10.1007/s12013-014-0083-5

**Published:** 2014-06-28

**Authors:** Wenjing Lan, Shengbang Wan, Weiqing Gu, Heyong Wang, Songwen Zhou

**Affiliations:** Department of Medical Oncology, Shanghai Pulmonary Hospital, School of Medicine Cancer Institute, Tongji University, No 507 Zhengmin Road, Shanghai, 200433 China

**Keywords:** Shikonin, Lung adenocarcinoma cell, Cell proliferation, Cell apoptosis, Cell cycle

## Abstract

Shikonin, a natural naphthoquinone isolated from a traditional Chinese medicinal herb, can exert inhibitory effect on tumor cell growth. However, little has been known concerning the effect of shikonin on lung adenocarcinoma cell and underlying mechanisms. In the present study, we investigated the effect of shikonin on the proliferation, cell cycle arrest, and apoptosis in human lung adenocarcinoma cells. We found that shikonin significantly suppressed the proliferation of lung adenocarcinoma cells compared with control in dose- and time-dependent manner (*P* < 0.05). In the meantime, our results showed that shikonin markedly increased the proportion of A549 cells at stage G1 as well as induced apoptosis in A549 cells. Furthermore, suppressed CCND1 and elevated caspase3 and caspase7 expression levels at mRNA were found in this study, indicating that shikonin may inhibit the growth of lung adenocarcinoma cell by changing cell cycle and promoting cell apoptosis through the regulation of CCND1, caspase3, and caspase7. Although more studies are needed, this study suggests that shikonin has the potential to be used as an anti-cancer agent in the treatment of lung adenocarcinoma.

## Introduction

Lung cancer will remain the leading cause of cancer-related death until 2030 [1], which is responsible for 1.3 million deaths every year worldwide. Every year, 1.5 million patients are diagnosed to have lung cancer, with 80–85 % of cases having non-small cell lung cancer (NSCLC) including lung squamous cell carcinomas, lung adenocarcinoma, and large cell carcinoma [2–6].

Although there are advances in therapeutic strategies, the prognosis of lung cancer remains poor with a 5-year survival rate of only 15 %. Surgery is the most effective option for patients with lung cancer. However, although majority of patients with lung cancer are diagnosed at late stage, only 30 % of them are suitable for curative resection. Therefore, chemotherapy and radiation are generally considered as the alternative options for these patients. Clinical evidence has showed that conventional radiation and/or chemotherapy are associated with serious side effects as well as poor efficacy [7]. It was reported that the effective rate in patients with advanced cancer was only 8.9–69 % after Gefitinib treatment [8]. Thus, an agent with high efficacy but few side effects is urgently needed in the clinical practice.

In recent years, compounds of Chinese traditional medicine have been investigated for their anti-cancer effects, and a number of compounds have been included in new strategies in cancer therapies [9]. Shikonin, derived from *Lithospermum erythrorhizon* (LE), has been used as medicine since the 5th century AD. It has been reported that Shikonin has a variety of biological activities including detoxification, anti-inflammatory effect, antitumor effect, and antiviral effect [7, 10–13].

A number of studies had investigated the anti-cancer effect of shikonin over the past four decades [14–21]. It was demonstrated that shikonin may inhibit tumor cell proliferation, induce tumor cell apoptosis, and change cell cycle. Previous studies found that shikonin may not only induce necroptosis in breast cancer cells, but also inhibit the proliferation of human colon cancer cell lines CCL229 [14, 22]. Meanwhile, studies found that shikonin may inhibit the growth of human skin cancer cell lines A431, human malignant melanoma cell line A375, and human prostate cancer cells [23–25]. Interestingly, a clinical trial found that shikonin mixture was effective in the treatment of patients with late-stage lung cancer, who were not qualified for surgery, radiotherapy, and chemotherapy [26]. However, the underlying mechanism of how shikonin exerts antitumor effect in lung cancer remains unclear.

Therefore, this study was designed to investigate the effect of shikonin on lung adenocarcinoma cell and the underlying mechanisms, which may provide a novel understanding to the antitumor effect of shikonin.

## Materials and Methods

### Materials and Reagents

Human lung adenocarcinoma cancer cell line A549 was obtained from the central lab of pulmonary hospital affiliated to Tongji University (Shanghai, China), which was cultured in Dulbecco’s modified Eagle’s medium (DMEM GIBCO, USA) supplemented with 10 % fetal bovine serum without mycoplasma (FBS, Sigma), penicillin (100 μg/ml), and streptomycin (100 μg/ml) from Qilu Pharmacy (Shandong, China). A549 cells were then cultured at 37 °C with 5 % CO_2_ in the incubator. Culture solution was replaced every 2 days. Cell morphology and vitality were observed by invert microscope (OLYMPUS, JAPAN).

### Cell Morphology

A549 cells at the logarithmic growth phase were seeded in 6-well tissue culture plates (COSTAR, USA) at appropriate concentration of (1–4) × 10^4^/ml and cultured at 37 °C with 5 % CO_2_ on the night. The next day, cells were exposed to various concentrations (0, 0.5, 1.0, 2.0, 4.0, and 8.0 μM) of shikonin from Sigma and then cultured at a temperature of 37 °C with 5 % CO_2_ for 24 h. The morphology of A549 cells was observed by invert microscope at 24 h, and photographs of cells were taken by single-lens reflex camera (OLYMPUS, JAPAN). Three fields of each plate were selected for taking the photograph.

### Cell Viability

Cell proliferation was measured by colorimetric [3-(4,5-dimethylthiazol-2-yl)-2,5-diphenyltetrazolium bromide] (MTT) assay. Briefly, A549 cells were cultured in DMEM medium supplemented with 10 % FBS at 37 °C with 5 % CO_2_ and digested in a logarithmic growth phase with 0.02 % EDTA (Beyotime, China) and 1× trypsin (Beyotime, China). The number of A549 cells was counted by cell counting plate and seeded into 96-well tissue culture plates (COSTAR, USA) at a density of (3–5) × 10^4^/ml with 100 μl/well, and cultured at 37 °C with 5 % CO_2_ on the night. The next day, cells were exposed to various concentrations of shikonin (0, 0.5, 1.0, 2.0, 4.0, and 8.0 μM) and then cultured at a temperature of 37 °C with 5 % CO_2_ for 24, 48, and 72 h, respectively. Each concentration was set in four wells. After cultured for 24, 48, and 72 h, 3-(4,5-dimethylthiazol-2-yl)-2,5-diphenyltetrazolium bromide (MTT, Gibco., American) solution was added into the 96-well tissue culture plates with 20 μl (5 mg/ml). The 96-well plates were centrifuged for 10 min at 1,000 rpm using centrifuge 5810R (Eppendorf, USA), and the supernatant was removed after culturing at 37 °C with 5 % CO_2_ for 4 h. 200 μl Dimethyl sulfoxide (DMSO, Gibco., American) was added into each well, and the well was shaken slowly in swing bed for 20 min. Optical density (OD) value was measured by enzyme-linked immune monitor (EPSON, China) at the wavelength of 530 nm. The inhibition ratio was set to 1- (OD/the mean inhibition ratio of control group). Each experiment was repeated 3 times.

### Cell Apoptosis

Apoptotic cells were measured using Annexin V/PI double dye kit. A549 cells in the logarithmic growth phase were seeded in 6-well tissue culture plates at the concentration of (3–5) × 10^5^/well and cultured at 37 °C with 5 % CO_2_ on the night. The next day, different concentrations (0, 0.5, 1.0, 2.0, 4.0, and 8.0 μM) of shikonin were added into the 6-well tissue culture plates. After cultured at 37 °C with 5 % CO_2_ for 24 h, A549 cells were digested with 0.02 % EDTA and 1× trypsin, suspended into single cell and centrifuged for 5 min at 1,000 rpm, and the supernatant liquid was discarded. The cells were suspended again with 1 ml of precooled 1× PBS (phosphate-buffered saline); the number of cells was counted at number <1 × 10^5^, centrifuged for 5 min at 1,000 rpm again, and finally collected. Binding buffer, PI (Annexin V/PI double dye kits, BIPEG), was added according to the protocol of apoptosis kit. Cell apoptosis was analyzed by flow cytometry (FCM, Becton–Dickinson, San Jose, CA, USA).

### Cell Cycle Distribution

Cell cycle distribution was determined by staining DNA with propidium iodide (PI). The A549 cells in the logarithmic growth phase were seeded in 6-well tissue culture plates at the concentration of (3–5) × 10^5^/well and cultured at 37 °C with 5 % CO_2_ on the night. The next day, different concentrations (0, 1.0, 4.0, and 8.0 μM) of shikonin were added into the 6-well tissue culture plates. After cultured at 37 °C with 5 % CO_2_ for 24 h, A549 cells were centrifuged for 5 min at 1,000 rpm, and the supernatant liquid was removed. The cells were rinsed with 1 ml of precooled 1 × PBS twice and then collected. 1 ml of 75 % alcohol was added for a night at the temperature of 4 °C. The next day, after centrifugation for 5 min at 1,000 rpm again, alcohol was removed, and the cells were washed twice with 1× PBS, and 1 ml PI was added into the cell. Then the cells were incubated in dark at 37 °C for 60 min. Cell cycle was analyzed by flow cytometry (FCM, Becton–Dickinson, San Jose, CA, USA), and the percentage of cells in the different phases of cell cycle was analyzed by FlowJo 7.6 software.

### RNA Extraction and Quantitative Real-Time PCR (RT-PCR)

A549 cells were seeded and grown to half confluence in six-well plates, and different concentrations (0, 1.0, 4.0, and 8.0 μM) of shikonin were added for 12 h. Total RNA in each group of cells was extracted with TRNzol-A+ reagent (Tiangen, Beijing, China). The concentration of RNA was determined by measuring the absorption in a spectrophotometer (NANODROP 2000, Thermo). The first cDNA strand was synthesized by TIANScript RT Kit (Tiangen, Beijing, China) and random primer from 1 µg total RNA according to the protocol. The RT reaction was initiated at 42 °C for 30 min, inactivated at 95 °C for 5 min, and then cooled at 16 °C for 5 min. Quantitative RT-PCR was performed using a SYBR^®^ Premix Ex Taq Mix (Takara Biotechnology Co., Ltd., Dalian, China) with forward and reverse primers for cyclin D1 (CCND1), cyclin D2 (CCND2), caspase3, caspase7, and β-actin. All primers were purchased from Invitrogen. The primer sequences were as follows:

CCND1: Forward primer: 5′-GCGAGGAACAGAAGTGC-3′,
Reverse primer: 5′-GAGTTGTCGGTGTAGATGC-3′;
CCND2: Forward primer: 5′ AATTCCTCCTCAATAGCCTGCAGCA-3′,
Reverse primer: 5′-GGATATCGACCTGTGAGAATTC-3′;
Caspase3: Forward primer: 5′-ACATGGCGTGTCATAAAATACC-3′,
Reverse primer: 5′-CACAAAGCGACTGGATGAAC-3′;
Capase7: Forward primer: 5′-GTCTCACCTATCCTGCCCTCAC-3′,
Reverse primer: 5′-TTCTTCTCCTGCCTCACTGTCC-3′.

PCRs were carried out in an ABI 7500 Real-Time PCR system (Applied Biosystems). The samples were incubated at 95 °C for 10 min, followed by 40 cycles at 95 °C 15 s and 60 °C for 1 min. When the cell cycling was completed, melting curve analysis was performed to establish the specificity of the PCR product. The cycle threshold (Ct) values were determined for the amplification of cyclin D1 (CCND1), cyclin D2 (CCND2), caspase3, caspase7, and β-actin. ΔCt was calculated by subtracting the Ct value for β-actin from the Ct value for each target gene. The expression of the target genes was normalized according to that of β-actin. The relative fold increase (RFI) was calculated by the ΔCt for treated and control cells using the following equation: ∆Ct = Ct (gene) – Ct (β − actin). The ΔΔCt value was then determined by subtracting the ΔCt value of the treated cells from the ΔCt value of the control cells and was used to calculate the RFI for the target gene using the following equation: RFI = 2^−ΔΔCt^. Each experiment was performed in duplicate and repeated three times.

### Western Blotting

Western blotting was performed according to previous studies [27, 28]. A549 cells were seeded and grown to half confluence in six-well plates, and different concentrations (0, 1.0, 4.0, and 8.0 μM) of shikonin were added for 12 and 24 h. Cells were washed with cold PBS, and proteins were extracted by radioimmunoprecipitation assay (RIPA) buffer (50 mM Tris–HCl, pH 7.4, 150 mM NaCl, 1 % NP-40, 0.25 % sodium deoxycholic acid, 1 M EDTA, 1 mM Na3VO4, 1 Mm NaF, and protease inhibitors cocktail). The levels of proteins were measured by BCA protein assay kit. The proteins were separated by electrophoresis on SDS-PAGE gel and electrotransferred onto a Hybond enhanced chemiluminescence (ECL, Beyotime, China) transfer membrane (Amersham Pharmacia, Piscataway, NJ). Then they were blocked with 5 % non-fat milk obtained from Guang Ming (Shanghai, China) in TBST solution [20 mM Tris–HCl (pH 7.6), 135 mM NaCl, and 0.1 % Tween 20], and probed with antibodies of CCND1, CCND2, caspase3, caspase7, PARP, cleaved PARP, and GAPDH for overnight. After they were washed for three times with TBST and incubated with secondary anti-mouse or rabbit antibodies (Sigma) for 2 h, GAPDH was measured by mouse monoclonal antibody at a dilution of 1:1,000 (Cell Signaling Technology Inc.), while others were measured by rabbit monoclonal antibody at a dilution of 1:1,000. Traditional film was used to detect the expression levels of proteins.

### Statistical Analysis

All the data were analyzed by SPSS 19.0, which were presented as mean ± SEM. One-way ANOVA and unpaired, two-tailed Student’s *t* test were used to analyze the data among the groups. *P* < 0.05 was considered as the significant difference.

## Results

### Impact of Shikonin on Cell Morphology

**Fig. 1:**
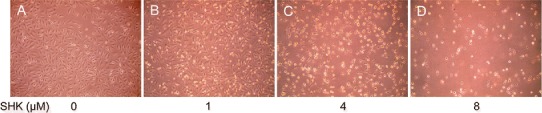
The effect of shikonin on the morphology of A549 cells. **a** Control group; **b** Cells were treated with shikonin at the concentration of 1.0 μM for 24 h. The number of A549 decreased and the morphology of A549 obviously changed; **c** Cells were treated with shikonin at the concentration of 4 μM for 24 h. The number of A549 obviously decreased and the morphology of A549 is different from that of the control with weak adherence; **d** Cells were treated with shikonin at the concentration of 8 μM for 24 h. Many cells died, and cell debris were found in substrate

A549 cells were well-grown in the same ship and in the form of fusiform or Polygon with rich cytoplasm in the control group. Compared with control, the number and morphology of A549 were obviously changed after the shikonin treatment at the concentration of 1.0 μM for 24 h. After the treatment for 24 h at the concentration of 1.0 μM, the number of A549 decreased obviously; at the same time, the morphology and the size of A549 were changed with weak adherence. Furthermore, when treated with 2.0 μM shikonin for 24 h, the number of cells drops sharply with different shapes. After 4.0 μM shikonin treatment for 24 h, the number of cells decreased dramatically, and the cell nucleus underwent pycnosis with decreased cell plasma and vacuolus in cytoplasm in the high power field of cytoplasm, which were easy to be taken off from the wall with solidification content and weak light-admitting quality. Finally, when treated with shikonin 8.0 μM for 24 h, many dead cells and cell debris were identified in the substrate besides decreased cell number and changed morphology. The morphological changes of A549 cells in the control and treatment groups at different shikonin concentrations are shown in Fig. 1.

### Proliferation Inhibition of Shikonin on A549

**Fig. 2:**
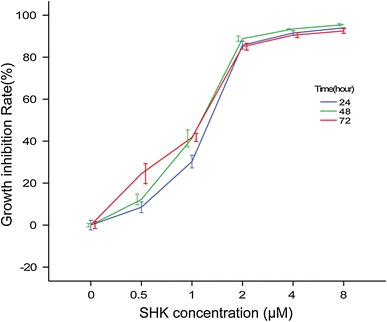
Shikonin inhibited A549 cells proliferation in a dose-dependent and time-dependent manner. *SHK* shikonin

As can be seen in Fig. 2, the inhibition ratio of A549 at all concentration groups (0.5, 1.0, 2.0, 4.0, and 8.0 μM) for 24, 48, and 72 h were all significantly higher than that of the control (*P* < 0.05 or 0.01). Statistical difference in the inhibition rate of A549 was also identified among the same concentrations of shikonin at different times, except between 24 and 48 h. Therefore, the inhibition of shikonin to A549 cells proliferation was dependent on both dose and time. On one hand, this proliferation inhibition was enhanced with the increase of shikonin concentrations. On the other hand, the proliferation inhibition was also enhanced with the increase of treatment duration.

### Apoptosis Induced by Shikonin

**Fig. 3:**
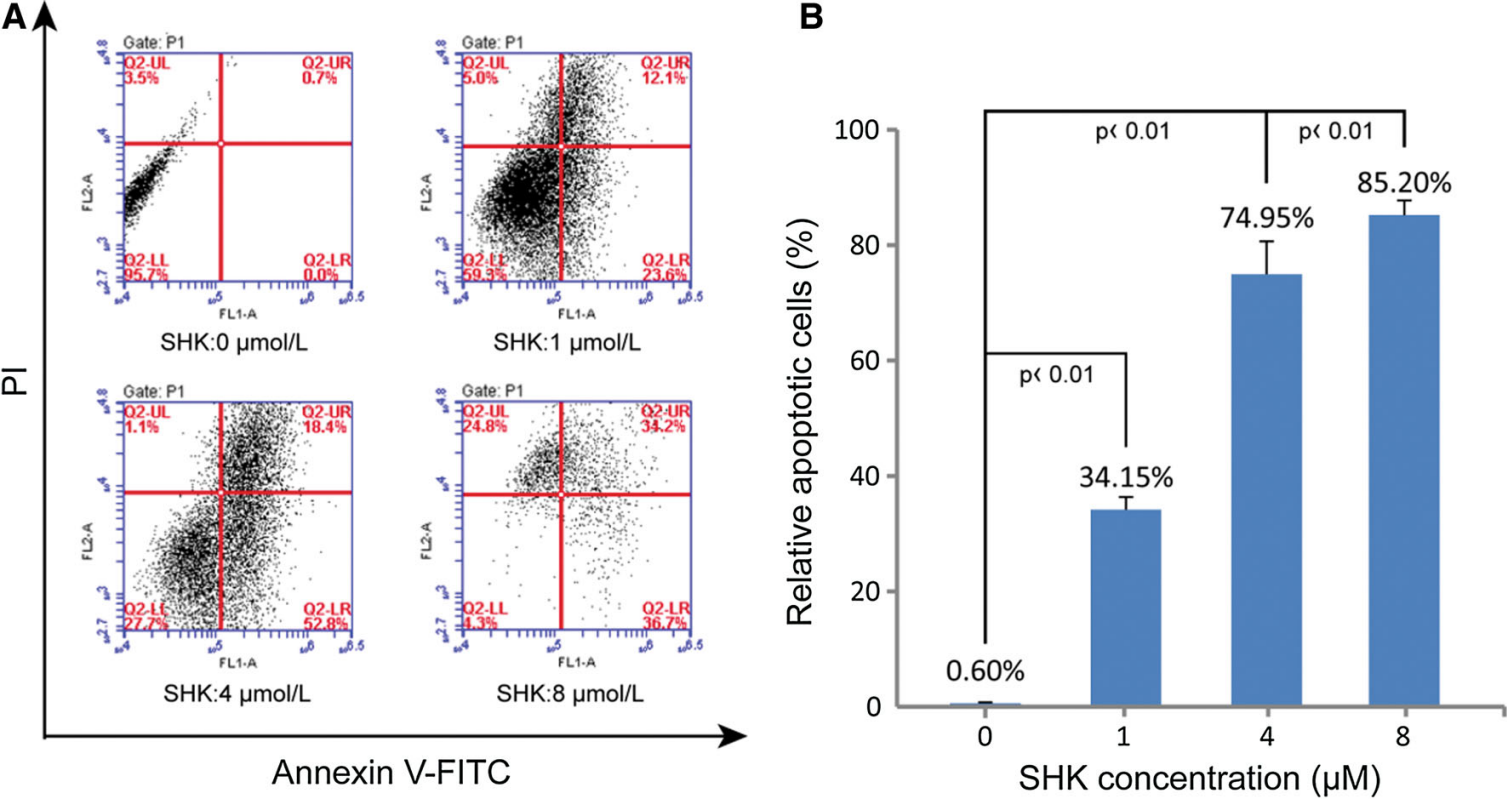
The effect of shikonin on the apoptosis of A549 cells. **a** Representative images of the flow cytometry analysis. Cells in the lower right quadrant (Q2-LR) indicate the percentage of Annexin-positive, early apoptotic cells. Cells in the lower left quadrant (Q2-LL) indicate the percentage of Annexin-negative/PI-negative, viable cells. Cells in the *upper right* quadrant (Q2-UR) indicate the percentage of Annexin-positive/PI-positive, late apoptotic cells. Cells in the *upper left* quadrant (Q2-UL) indicate the percentage of PI-positive, necrotic cells; **b** *Graphic* representation of apoptotic levels (early plus late apoptosis) after shikonin treatment at different concentrations for 24 h. Data were expressed as mean ± SEM

In Fig. 3, the apoptosis rate of A549 was 0.6 ± 0.141, 8.25 ± 0.495, 34.15 ± 2.192, 59.1 ± 0.424, 74.95 ± 5.728, and 85.2 ± 2.546 %, respectively, after shikonin treatment at different concentrations (0, 0.5, 1.0, 2.0, 4.0, and 8.0 μM) for 24 h. The apoptosis rate increased accordingly with the increase of shikonin concentrations. The apoptosis rate of A549 cells treated with shikonin at different concentrations (0.5, 1.0, 2.0, 4.0, and 8.0 μM) was significantly higher than that of the control (*P* < 0.05). Thus, shikonin may induce the apoptosis of human lung adenocarcinoma cell line A549.

### Shikonin Induces Cell Cycle Arrest at the G0/G1 Phase in A549 Cells

**Fig. 4:**
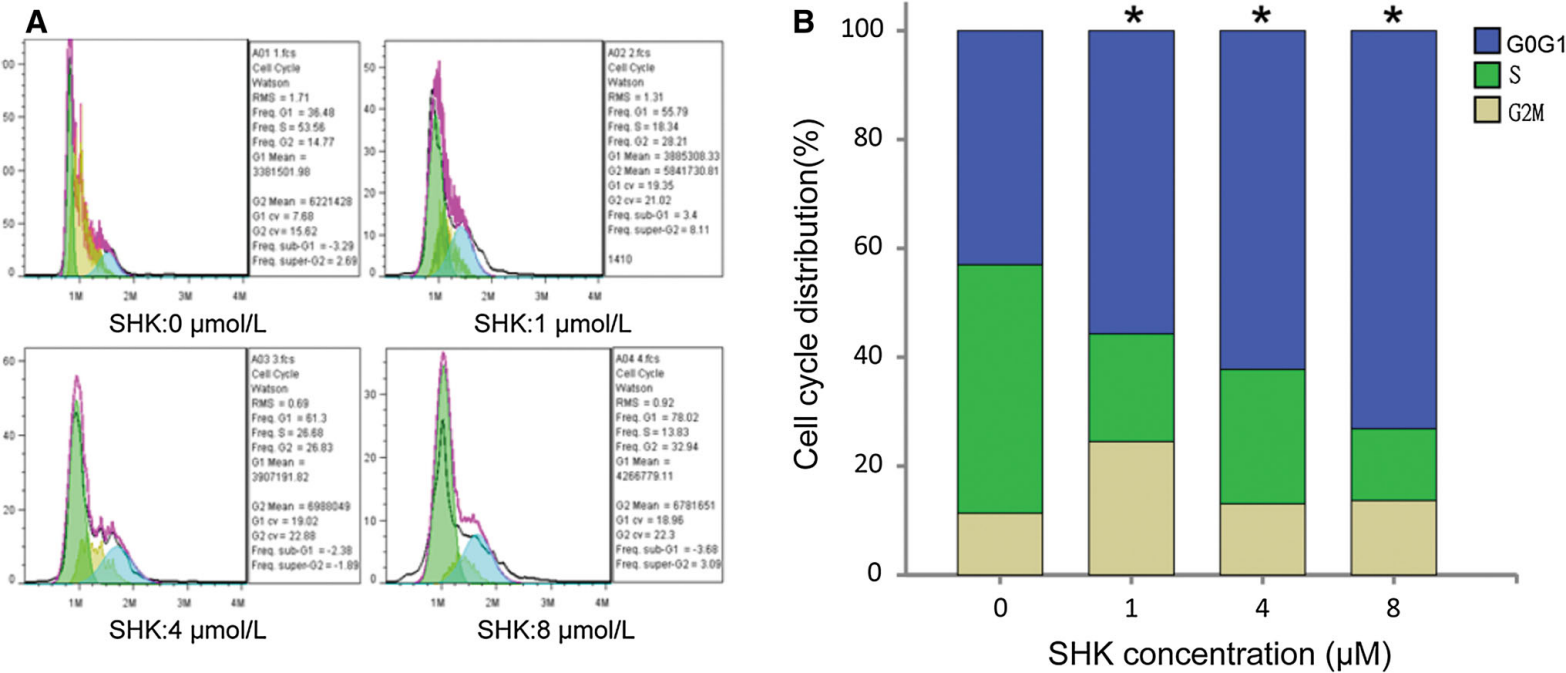
The effect of shikonin on cell cycle of A549 cells. **a** Flow cytometric analysis on A549 cell line to determine the stage of cells after incubation with shikonin at 0, 1.0, 4.0, and 8.0 μM. **b** The percentages of cells in each phase of cell cycle are shown. With the increase of shikonin concentrations, the proportion of G0/G1 stage increased as shown in the *blue column*. *P < 0.01 vs control

As shown in Fig. 4, the proportion of A549 cells in stage G1 increased with the increase of shikonin concentrations. The proportions of A549 cells in G1 stage were 43 ± 1.348, 55.697 ± 1.852, 62.233 ± 0.862, and 73.127 ± 4.489 %, respectively, at different concentrations of shikonin (0, 1.0, 4.0, and 8.0 μM). Furthermore, the proportions of A549 cells in G1 stage treated with shikonin at the concentrations of 1.0, 4.0, and 8.0 μM were all significantly higher than that of the control (*P* < 0.05). Therefore, G1 stage was blocked by shikonin.

### Effects of Shikonin on the Expression levels of Cell Cycle-Related Genes and Apoptosis-Related Genes

**Fig. 5:**
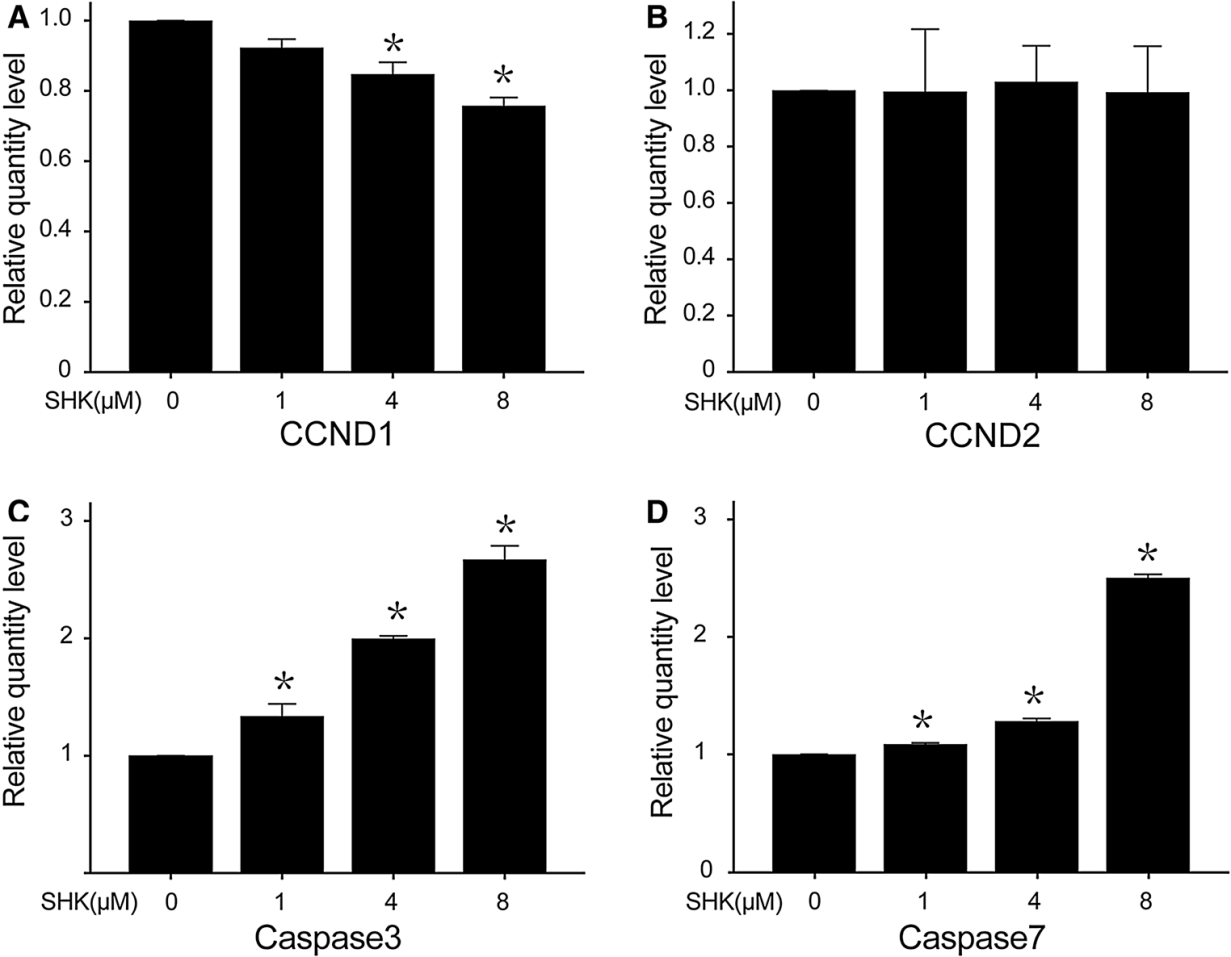
Expression levels of apoptotic genes and cycle-related genes by RT-PCR. **a** The levels of CCND1 markedly decreased when exposed to various concentrations (0, 1, 4, and 8 μM) of shikonin. **b** The levels of CCND2 did not change with the increase of shikonin concentration. **c** The level of caspase3 markedly increased with the increase of shikonin. **d** The level of caspase7 markedly increased with the increase of shikonin. *P < 0.05 vs control

As can be seen in Fig. 5, the level of CCND1 significantly decreased when exposed to different concentrations of shikonin. With the increase of shikonin concentrations for 12 h, the level of CCND1 has a significantly declined trend, but no such trend was identified for CCND2 levels.

On the other hand, the levels of caspase3 and caspase7 also have a rising trend. The levels of caspase3 and caspase7 significantly increased when treated with different concentrations of shikonin.

### Shikonin Induces Apoptosis Through Regulating Cell Cycle-Related Proteins and Increasing Caspases Activation in A549 lines

**Fig. 6:**
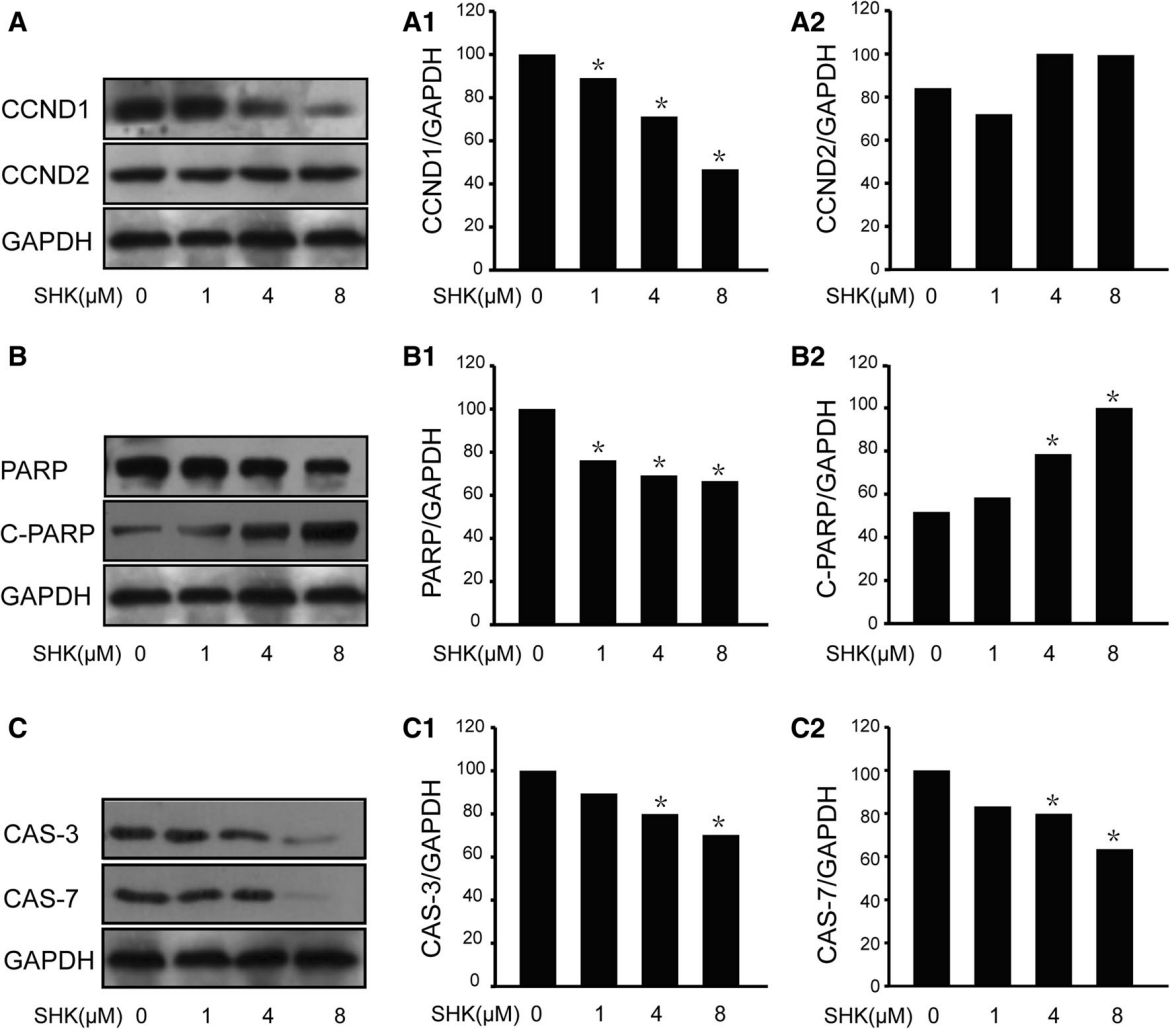
Expression levels of apoptotic genes and cycle-related genes by Western blotting. **a** The levels of CCND1 and CCND2. **a1** The expression level of CCND1 reduced with the increase of shikonin. **a2** The level of CCND2 remained stable whatever was the concentration of shikonin. **b** The levels of PARP and cleaved PARP. **b1** The level of PARP was markedly declined with the increase of shikonin concentrations. **b2** The level of cleaved PARP increased. **c** The levels of caspase3 **c1** and caspase7 **c2**. The expression levels of caspase3 and caspase7 were decreased with the increase of shikonin concentrations. *P < 0.05 vs control

As shown in Fig. 6, the expression of CCND1 declined in A549 cell lines with the increase of shikonin, but no change was found in CCND2. At the same time, caspases including caspase3, caspase7, and PARP which participate in the classical apoptosis signal transduction pathways were also activated. Thus, shikonin may induce apoptosis through regulating cell cycle-related proteins and increasing caspases activation in A549 lines.

## Discussion

In this study, we found that shikonin exerted anti-cancer effects through suppressing cell proliferation, inducing cell apoptosis, and affecting cell cycle. Some phytochemicals from Chinese medicinal herbs may have antitumorigenic activities through inducing the apoptosis of cancer cells, which was demonstrated by several previous studies [29, 30]. Sankawa et al. found that shikonin and a range of simple derivatives completely inhibited tumor growth in mice at a dose of 5–10 mg/kg/day [31, 32]. The mechanisms of apoptosis of cancer cells induced by Chinese medicinal herbs may be related with the activation of not only caspases (cysteinyl aspartate-specific proteases), but also bcl-2 family [33, 34].

The apoptosis of cancer cells has two phases: a commitment to cell death and an execution phase featured with dramatic morphological changes in cell structure [35]. Apoptosis is a major form of cell death, characterized initially by a series of stereotypic morphological changes. It was found in our study that the low concentrations of shikonin may change the number and morphology of A549 lines, and higher concentrations of shikonin may even induce the apoptosis of many cells.

The inhibition of shikonin to A549 cells proliferation was dependent on both dose and time. Shikonin may inhibit the growth rate of A549 cell lines by the following two mechanisms: inducing cell apoptosis and changing cell cycle. Shikonin may induce cell apoptosis through the activation of caspase family, such as caspase3, caspase7, and PARP. Caspases (Cysteinyl Aspartate-Specific Proteases) are groups of cysteine-containing proteolytic enzymes produced by the cells of living organism. A specific caspase-activating complexes—apoptosome, mediating caspase9 activation, may interact with the apoptotic protease-activating factor-1 (Apaf-1) adaptor in the presence of cytochrome [36, 37]. In our study, caspase3, caspase7, and PARP were all activated, which was proved by Western blotting. Also, the mRNA levels of caspase3 and caspase7 were increased by performed RT-PCR. This result indicated that shikonin may induce cells apoptosis both at protein level and mRNA level. Additionally, shikonin influenced the cell cycle by increasing the proportion of G0/G1 stage, allowing cells to stay in the G0/G1 phase—which made it difficult to enter into the next period and reduce the ratio of S stage and G2/M stage. Therefore, the cell mitosis is abnormal.

On the other hand, cell-cycle regulatory proteins such as cyclin D family may play important roles in modulating the cell cycle. CCND1, a cell-cycle regulator and a candidate proto-oncogene, is essential for the progression through G1 phase [38]. In this study, CCND1 presented a decline in trend with the increase of shikonin concentration, but the expression of CCND2 remained stable, which demonstrated that shikonin may change A549 cells cycle at both transcription and translation levels.

The results of this study suggested that shikonin may be a novel and attractive therapeutic candidate for tumor treatment in clinical practice. However, further research is needed to identify the other mechanisms of shikonin on A549 cells. Additionally, randomized controlled trial was also needed to demonstrate the efficacy and safety of shikonin in NSCLC patients.

In conclusion, shikonin may inhibit the growth of lung adenocarcinoma cell by changing the cell cycle and promoting the cell apoptosis through the regulation of CCND1 and caspase family (caspase3 and caspase7). Shikonin may be a potential drug in the treatment of NSCLC.
